# INTEREST GROUP SESSION—DISASTERS AND OLDER ADULTS: HARNESSING THE POWER OF SOCIAL NETWORKS DURING DISASTERS TO FACILITATE PREPARATION AND RECOVERY

**DOI:** 10.1093/geroni/igz038.1434

**Published:** 2019-11-08

**Authors:** Judith Robertson R Phillips, Katie E Cherry

**Affiliations:** 1 California State University San Marcos, San Marcos, California, United States; 2 Louisiana State University, Baton Rouge, Louisiana, United States

## Abstract

With the increasing occurrences of disasters throughout the world, researchers, communities, and organizations have become interested in how the use of social networks during and after a disaster can ease the psychological recovery of older adults who are affected by traumatic disaster events such as hurricanes and wildfires. This symposium will focus on the power of social networks and highlight the importance of preparedness, informal and formal groups, and interventions to assist recovery of older adults. First, Dr. Judith Phillips will present data on how informal and formal social network groups affected the psychological well-being of older adults who experienced exposure to multiple wildfires. Second, Dr. Denise Eldemire-Shearer will address how both formal and informal social network groups in Jamaica are informed and mobilized to provide support to older adults on the island during hurricanes and other water-related disasters. Third, Dr. Lisa Brown will introduce the Skills for Psychological Recovery intervention and provide an overview and modifications needed when using with older adults; she will also demonstrate an exercise. Fourth, Dr. Debra Dobbs will present themes examining the role of community engagement in hurricane preparedness which were gathered from focus groups and interviews with assisted living administrators in Florida. Lastly, Dr. Mary Helen McSweeney-Feld will address how voluntary organizations active during disasters aid older adults with disaster relief efforts. Together these presenters will provide evidence of the power of various social networks that will lessen the vulnerability of older adults after disasters.

